# TCMSF: A Construction Framework of Traditional Chinese Medicine Syndrome Ancient Book Knowledge Graph

**DOI:** 10.1055/a-2590-6348

**Published:** 2025-05-15

**Authors:** Ziling Zeng, Lin Tong, Bing Li, Wenjing Zong, Qikai Niu, Sihong Liu, Lei Zhang, Jialun Wang, Siqi Zhang, Siwei Tian, Jing'ai Wang, Wei Zhang, Huamin Zhang

**Affiliations:** 1 Materia Medica Research Center, Institute of Chinese Materia Medica, China Academy of Chinese Medical Sciences, Beijing, People's Republic of China; 2 The Ancient Book Resources Research Office, Institute of Information on Traditional Chinese Medicine, China Academy of Chinese Medical Sciences, Beijing, People's Republic of China; 3 Materia Medica Digital Intelligence Center, Institute of Chinese Materia Medica, China Academy of Chinese Medical Sciences, Beijing, People&s Republic of China; 4 Integrated Research Center for Chinese Materia Medica, Institute of Chinese Materia Medica, China Academy of Chinese Medical Sciences, Beijing, People's Republic of China; 5 Department of Special Collections Research and Development, Institute of Information on Traditional Chinese Medicine, China Academy of Chinese Medical Sciences, Beijing, People's Republic of China; 6 Institute of Basic Theory for Chinese Medicine, China Academy of Chinese Medicine Science, Beijing, People's Republic of China

**Keywords:** TCM, knowledge organization, knowledge graph, ancient book, relationship extraction

## Abstract

**Background:**

Syndrome is a unique and crucial concept in traditional Chinese medicine (TCM). However, much of the syndrome knowledge lacks systematic organization and correlation, and current information technologies are unsuitable for TCM ancient texts.

**Objectives:**

We aimed to develop a knowledge graph that presents this knowledge in a more orderly, structured, and semantically oriented manner, providing a foundation for computer-aided diagnosis and treatment.

**Methods:**

We developed a construction framework of TCM syndrome knowledge from ancient books, using a pretrained model and rules (TCMSF). We conducted fine-tuning training on Enhanced Representation through Knowledge Integration (ERNIE), Bidirectional Encoder Representation from Transformers pretrained language models, and chatGLM3–6b large language models for named entity recognition (NER) tasks. Furthermore, we employed the progressive entity relationship extraction method based on the dual pattern feature combination to extract and standardize entities and relationships between entities in these books.

**Results:**

We selected Yin deficiency syndrome as a case study and constructed a model layer suitable for the expression of knowledge in these books. Compared with multiple NER methods, the combination of ERNIE and Conditional Random Fields performs the best. By utilizing this combination, we completed the entity extraction of Yin deficiency syndrome, achieving an average F1 value of 0.77. The relationship extraction method we proposed reduces the number of incorrectly connected relationships compared with fully connected pattern layers. We successfully constructed a knowledge graph of ancient books on Yin deficiency syndrome, including over 120,000 entities and over 1.18 million relationships.

**Conclusion:**

We developed TCMSF in line with the knowledge characteristics of ancient TCM books and improved the accuracy of knowledge graph construction.

## Introduction

Syndrome, a unique concept in traditional Chinese medicine (TCM), is the general term for a series of related symptoms. Yin deficiency syndrome, as one of the two most classic basic syndromes in TCM, has guiding significance for the diagnosis and treatment of diseases in TCM. However, the lack of systematic organization has hindered the effective utilization of knowledge related to the Yin deficiency syndrome and its close relationships, especially in ancient literature.

The knowledge graph (KG) is a new model of massive knowledge management and organization in the context of the big data era. Transforming TCM knowledge into graph form for storage is more conducive to the dissemination, preservation, and innovative development of TCM through modern information methods. The existing knowledge maps in the field of TCM mostly focus on diseases, with fewer knowledge maps constructed based on syndromes. They are mostly constructed from modern literature as data sources, which differs considerably from the knowledge system of ancient literature.

Therefore, in this study, we used ancient Chinese medicine books as the data source and selected Yin deficiency syndrome as an example to explore and develop knowledge representation methods suitable for the semantics of ancient Chinese medicine books and key technologies for constructing a KG of TCM syndromes.

### Related Works

TCM KGs are mostly built around diseases, and a few scholars have constructed KGs centered on TCM syndromes based on the dialectical knowledge of modern Chinese medicine literature. For example, Guo et al 1 and Zhou et al 2 have constructed a TCM syndrome differentiation knowledge map based on the knowledge of syndrome differentiation in TCM textbooks.

Information extraction is an essential part of building a large-scale knowledge map, which includes named entity recognition (NER) and relationship extraction (RE). At present, the research on NER in the field of TCM is mainly focused on modern literature, especially electronic medical records 3 4 5 and modern medical cases; 6 limited attention has been paid to ancient TCM texts. Recent studies on NER in ancient TCM books highlight a shift from the traditional Conditional Random Field (CRF) model, which integrates lexical and semantic features, toward advanced deep learning approaches that leverage domain-specific pretrained language models and structural character embeddings, thereby improving the effectiveness of NER tasks. 7 8 9 However, the accuracy of NER for ancient TCM books remains low compared with that for modern literature, perhaps because ancient books are written in classical Chinese, and their parts of speech, collocation, and usage habits differ significantly from those in modern Chinese. The corpus is richer than ancient books, with more keywords and characteristic words for guiding recognition. In addition, the context is closer than the literature on TCM theory, and the method of extracting entities by relying on context information has advantages. The emergence of large language models (LLMs) has become a new trend in natural language processing; however, their application to NER tasks in TCM texts, particularly ancient literature, remains underexplored.

RE has always been a challenging task in the field of Natural Language Processing. 10 Currently, there are primarily rule-based or deep learning-based methods for RE in the construction of TCM KGs. The former generally adopts methods such as pattern layer-based RE, keyword-based template matching, or a combination of the two. In addition to using different deep learning models, the latter is also divided into the pipeline method (i.e., NER and RE are conducted in two steps) and the joint model extraction method based on the RE process. Currently, joint models are the main method. For example, Wang et al have proposed a new joint model that involves a multihead attention layer and adversarial training method to extract five types of entities and eight types of relationships. 11 Sun et al have proposed a joint model leveraging a multihead mechanism based on an existing pretrained model to extract three types of entities and two types of relationships. 4

The deep learning-based RE method is based on the training of relationship classification using triples of annotated relationships. The learned knowledge, such as grammar and semantics, is used for relationship recognition and classification, and it performs well in general fields and modern Chinese texts. However, deep learning may lead to insufficient semantic understanding or difficulty in handling ambiguity due to ignoring language structure information, thereby affecting the performance of the model. Recent studies have sought to address these limitations by integrating syntactic analysis into deep learning frameworks. For instance, by modeling joint entity-relation extraction as full shallow semantic dependency parsing, incorporating a second-order scoring module to exploit the relationships between entities and relations, and utilizing feature tagging methods based on a pretrained language model to enrich token representations, the performance of entity RE is improved. 12 However, many relationship instances are often expressed across multiple sentences, nested in documents with complex semantic structures, which is particularly prominent in ancient Chinese medicine books. Syntactic analysis is difficult to handle issues such as long-distance dependencies, nested structures, and complex semantic relationships. Deep learning cannot fully utilize entity relationships and subdomain relationship feature knowledge in the pattern layer that has been confirmed by domain experts and cannot have clear constraints on entity relationships. Especially in the field of TCM, the relationships between different types of entities in ancient literature are complex, with few prompts in original texts. Simultaneously, other constraints beyond the entity relationships require the participation of TCM experts to be understood. 13 In the general and biomedical fields, scholars have developed a relation semantic template or a unified framework based on event constraint information, constraining the process of relation classification and triplet extraction. 14 15 However, the relationship constraint information framework in the field of TCM ancient books is still being explored.

Given the current lack of construction of KGs for TCM ancient literature-related syndrome and scant research on knowledge representation methods for TCM ancient literature-related syndrome and key technologies for computer automatic construction of KGs, we extract the grammatical context and writing characteristics of TCM ancient literature to form a progressive entity RE method based on dual pattern feature combination. Combined with the pretrained model, we propose a construction framework of TCM syndrome ancient book KG based on the pretrained model and rules (TCMSF); on this basis, we construct a KG of TCM ancient literature with Yin deficiency syndrome.

## Methods

In this study, we designed TCMSF to achieve the extraction and standardization of entities and relationships related to the syndrome in TCM ancient literature. We selected Yin deficiency syndrome as an example to construct a KG of TCM ancient syndrome literature. We first derived the relevant knowledge of Yin deficiency syndrome from the TCM ancient literature database “Zhong Hua Yi Dian” and screened and supplemented knowledge. Subsequently, we constructed a KG schema layer of Yin deficiency syndrome with the expression characteristics of TCM syndrome knowledge. We then established an automatic tagging vocabulary, designed and implemented a system for tagging text entities and their relationships, and used it to conduct human–machine annotation of deep learning training data. We used training data to fine-tune ERNIE (Enhanced Representation through Knowledge Integration)/BERT (Bidirectional Encoder Representation from Transformers) + CRF and identify named entities. As a comparison, we also used GPT3.5 and ChatGLM for the entity recognition test. We, then, proposed a progressive entity RE method based on a dual pattern feature combination. Finally, neo4j was used to construct the knowledge map of Yin deficiency syndrome in ancient literature.

### Data Source

We first identified the connotation of “Yin deficiency syndrome,” clarified the research scope and data screening criteria of ancient books related to Yin deficiency syndrome, and finally chose 17 pathogenesis search terms related to “Yin deficiency syndrome” and 11 related treatment search terms. Using these 28 keywords, we retrieved 43,941 articles of ancient literature before 1,911 (including 1,911) in the text database “Zhong Hua Yi Dian.” Then, we used 14 inclusion and exclusion criteria to conduct screening, manually supplemented the previous and subsequent articles with incomplete semantics, manually disassembled the articles with errors, and manually proofread the mistakes and omissions in the text. Finally, a total of 21,568 pieces of high-quality text were obtained.

### Schema Layer Construction

According to the knowledge characteristics of Yin deficiency syndrome, combined with the existing recognized terminology classification system “Health informatics-Semantic network framework of the traditional Chinese medicine language system” (GB/T 38324-2019, ISO/TS 17938:2014) and “Traditional Chinese Medicine-Categories of traditional Chinese medicine clinical terminological systems” (ISO 19465:2017), the model layer of knowledge map of ancient books on Yin deficiency syndrome was constructed, and the core entity types and relationships were preliminarily extracted. In addition, we manually tagged entities by deleting entity types with extremely sparse data and adding entity types that were not covered. Finally, the model layer of the knowledge map of ancient books with Yin deficiency syndrome was determined by consulting experts.

The schema layer of the knowledge map of ancient books on Yin deficiency syndrome we developed contains 23 entity types related to yin deficiency syndrome: “disease,” “symptom,” “cause and mechanism of disease,” “herb,” “formula,” “constitution,” “physician,” “geographical name,” “therapeutic principle and method,” “tongue manifestation,” “pulse condition,” “nature of disease,” “location of disease,” “staging,” “joint formula,” “time,” “age,” “gender,” “prognosis,” “title of TCM ancient books,” “dynasty of book completion,” “year of book completion,” and “article ID.” Furthermore, the 29 types of entity relationships encompass: “treat,” “phenomenon express,” “include,” “occurrence,” “cause,” “influence,” “be related to,” “use,” “in,” “be applicable to,” “contraindicate,” “co-occurrence,” “record in,” “active in,” “discuss,” “consist of,” “the author is,” “be written in,” “basic formula is,” “the composition of basic formula is,” “add herb to basic formula,” “remove herb to basic formula” “add formula to basic formula,” “the basis for adding herb,” “the basis for removing herb,” “the basis for adding formula,” “cite,” “come from,” “article ID is,” and relationship attribute “dosage.” Among the schema layer, we set the entity type to “joint formula” so that the modification of herb and formula can correspond to the basis for modification one by one. We set “dosage” as the relationship attribute of “consist of” to correspond “formula” or “joint formula” to the dosage one by one.

### Named Entity Recognition

We used deep learning methods based on pretrained models to extract the content of ancient books related to Yin deficiency syndrome. The types of named entities extracted are the 23 types of Yin deficiency syndrome-related entities defined in the schema layer. The first 20 types of entities could be extracted by deep learning methods based on pretrained models, whereas the “dynasty of book completion” and “year of book completion” entities were extracted by matching the source ancient book names of the articles and the “Information Table of TCM Ancient Books” collated by our research group in the early stage. The ancient book articles were automatically numbered as “article ID” entities.

We needed to conduct fine-tuning training on the pretrained model for entity recognition in ancient books related to yin deficiency. The fine-tuning training was derived from 50% of the 21,568 ancient book text articles annotated by the human–computer combination. The human–computer combination annotation method we adopted consists of two steps. The first step is to automatically annotate the ancient book articles using string matching and regular expression methods based on self-built automatic annotation dictionaries and rules. The second step is to manually verify and supplement the automatic annotation results. The self-built automatic annotation dictionary contains the names of entities in the field of TCM and their classification names, derived from various terminology specifications with clear noun classification, TCM dictionaries, TCM textbooks, and online databases, such as “Traditional Chinese Medicine Clinical Diagnosis and Treatment Terminology,” “Traditional Chinese Medicine Dictionary,” and “Traditional Chinese Medicine Syndrome and Disease Dictionary.” We used text annotation software designed and developed by us based on Java language for manual verification and supplement annotation. It has three basic functions: entity annotation, relationship annotation, and entity standardization. It also has the advantages of ease of use, simple and flexible tag definition methods, and no impact on the reading experience of the original text after annotation. Before manual verification and supplement annotation, we developed a labeling rule manual, including the definition of entity types, the granularity of labeling, and corresponding examples. Subsequently, two to three rounds of repeated training and screening of manual labeling personnel were conducted to ensure that the labeling accuracy was greater than 80%.

Fine-tuning training for NER tasks based on pretrained is a supervised training process using a redefined entity classification output layer and labeled datasets based on a previously self-supervised pretrained model. As a comparison, we used Softmax function and CRF as the output layer of the pretrained model ERNIE/BERT for entity recognition fine-tuning training and prediction, and Precision, Recall, and F1 score as evaluation metrics for the training results. In addition, we also used the same test data to predict entity recognition in GPT3.5 of the open AI and ChatGLM of Tsinghua University.

Because entity recognition methods using deep learning technology cannot guarantee 100% accuracy and complete recognition of every entity, after using the pretrained model for entity recognition, we again imported the recognition results into text annotation software and manually corrected and supplemented the annotations to ensure the quality of the knowledge in the KGs.

### Relation Extraction

Although we defined the relationship between entity types in the schema layer, the expression of this relationship during instantiation depends on different contexts, syntax, and grammatical structures. For instance, we defined the relationship “cause” between “cause and mechanism of disease” and “pulse condition” in the schema layer, but not all entities under these two types of entities in the same piece of text have a “cause” relationship. We took a part of the text of an ancient book as an example, in which “soft pulse” and “slippery pulse” are both pulse entities, whereas “Yin deficiency of viscera” and “phlegm heat” are both “cause and mechanism of disease” entities. However, according to the context, we know that these represent two parallel combinations of “pulse” and “cause and mechanism of disease.” “Yin deficiency of viscera” causes “soft pulse,” whereas “Phlegm heat” causes “slippery pulse,” which can be connected into two triples <Yin deficiency of visceral, cause, soft pulse> and <phlegm heat, cause, slippery pulse > , whereas the “causing” relationship between “Yin deficiency of visceral” and “slippery pulse” and “phlegm heat” and “soft pulse” does not have a relationship expressed in this sentence and cannot be connected into a relational triplet. Therefore, we could not directly connect all entities in the articles according to the relations designed in the schema layer. Otherwise, many wrong relations would be stored in the database, resulting in incorrect and invalid use of the literature's information in the application.

The process of TCM syndrome differentiation and treatment is described in ancient books as follows. According to the patient's symptoms, signs, or confirmed diseases, it is necessary to identify the patient's syndrome, analyze the cause and mechanism of the patient's disease, and then discuss the methods and principles of treatment. Then, the combination of formula or herb for treatment is given (or the addition and subtraction of herb are conducted according to the patient's symptoms, signs, diseases, etiology and pathogenesis, treatment principles, and methods based on the existing prescription). In this process, multiple causes and disease mechanisms, symptoms, and signs (such as pulse condition or tongue manifestation), or entities of the same type as the treatment principle and treatment method have both a specific relationship and a “co-occurrence” relationship with other types of entities during the process of syndrome differentiation and treatment. In the treatment of medication, a basic formula (or a group of herbs) is accompanied by the addition and subtraction scheme of a basic formula and the basis of addition and subtraction, as well as a whole of the “co-occurrence” relationships relative to the other entities, aiming at the specific syndrome differentiation and method of treatment of the patient. Importantly, the process of syndrome differentiation and treatment method described in ancient books is not complete most of the time, and some parts may be missing, requiring the focus on the relationship between certain types of entities.

**Fig. 1:**
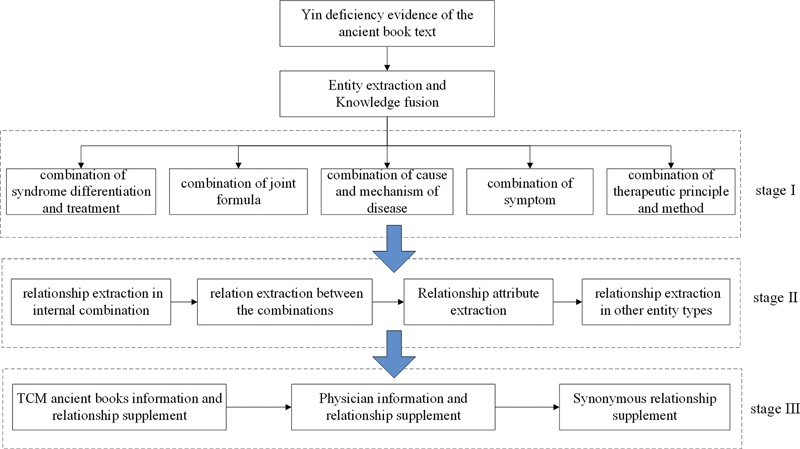
Process of relation extraction.

According to the analysis of the process of syndrome differentiation and treatment of TCM, we designed a RE process in three stages, as shown in Fig. 1 . In the first stage, we should model the combination of entities with “co-occurrence” relationships and the syndrome differentiation and treatment process, which means putting forward the framework of relationship representation.

A group of multiple entities of the same type that appear continuously in the text, including causes and mechanisms of disease, symptom, tongue manifestation, pulse condition, and therapeutic principles and methods, respectively, form a simple co-occurrence combination of similar entities, which are named the cause and mechanism of disease combination, symptom and sign combination, and therapeutic principle and methods combination.

A continuous co-occurrence of a basic formula entity or a group of herbs, together with the addition and subtraction scheme of the basic formula and the basis of its addition and subtraction, constitute an entity combination. To this end, we proposed a framework of “basic formula + joint formula (the basis for joint formula) + added herb (the basis for the addition of herb) + subtracted herb (the basis for the subtraction of herb)” to construct the entity combination named “combination of joint formula,” where each element entity jointly constitutes the combination, and the “ + ” represents the union, whereas the parentheses represent the correspondence between the basis and measure. The “basic formula” in the framework can be the first formula entity or the first group of continuous entities that appear in the text, and the “basic formula” element can appear only once, while other elements can appear multiple times. The “combination of joint formula” can be one or more formula entities that appear again after the “basic formula” appears, and there are obvious combination formula keywords in front of the entities.

The “basis of combination” can be the symptom, sign, disease, cause and mechanism of disease, therapeutic principle, and methods entity or combination on both sides of the “combination of joint formula.” Furthermore, “addition of herb” or “reduction of herb” can be one or more consecutive entities of herb with obvious addition or reduction keywords in the front, such as “add” and “remove.” In addition, some keywords represent replacing single or partial entities of herb in the “basic formula” with other entities of herb, such as “replace… For…,” “use… For…,” and so on, which can be divided into two processes: “addition of herb” and “reduction of herb.” “The basis for adding herb” and “the basis for reducing herb” are similar to “the basis for adding formula,” which can be the entity or combination of symptom, sign, disease, cause and mechanism of disease, and therapeutic principle and method, on both sides of the entity of “addition of herb” or “reduction of herb.”

Finally, all entities of a complete or incomplete syndrome differentiation and treatment process were combined into the combination of syndrome differentiation and treatment. Therefore, we proposed a second framework “disease/symptom or sign → cause and mechanism of disease → therapeutic principle and method → formula/herb,” named the combination of syndrome differentiation and treatment. If there are two or more of the four process elements, they can form a combination of syndrome differentiation and treatment, and “→” indicates the process direction of syndrome differentiation and treatment. The “disease/symptom or sign” in the framework can be an entity or combination of them. Because the entity of disease and the other three entities or combinations generally do not occur simultaneously, it is separated by “/” in the framework. The “cause and mechanism of disease” and “therapeutic principle and method” elements can be an entity or combination of the same type. “Formula/herb” is the final disposal plan in the process of syndrome differentiation and treatment, which can be formulas or a group of herb entities (both of which sometimes occur simultaneously) or a combination of formulas. Importantly, when using the second framework to construct the combination of syndrome differentiation and treatment, the principle of element uniqueness should be observed. More precisely, each element in the framework is unique, and two entity elements of the same type that do not form a combination, two entity combinations of the same type, independent entities of the same type, and entity combinations (the combination of symptom groups include symptoms and signs).

The first stage involves symptom, pulse condition, tongue manifestation, disease, cause and mechanism of disease, nature of disease, location of disease, therapeutic principle and method, and formula and herb, which are the most frequent and important entities in the process of syndrome differentiation and treatment of TCM.

In the second stage, using the relationship design of entity types in the schema layer, the relationships between entities, between combinations, between entities and combinations, and between these entities and other non-10 main types of entities extracted in the first stage are instantiated as relationship/attribute triples.

When determining the entity relationship in the cause and mechanism of disease combination, symptom combination, therapeutic principle, and method combination, two entities in the combination are connected by the “co-occurrence” relationship. When determining the entity relationship in the combination of the formula, we added a virtual entity of the “joint formula” type, which associates the entities in the combination with the entities of the joint formula. Their relationships are, respectively, expressed as “basic formula is,” “the addition of herb of basic formula,” “the basis of adding herb,” “the basis of joint formula,” “the reducing of herb of basic formula,” and “the basis for reducing herb.” If the “basic formula” is not due to a formula entity composition but a group of herb entities, the relationship between the joint formula entity and the herbs of the “basic formula” is “the composition of the basic formula.” The relationship between entities in the combination of joint formula and other entities outside the combination is indirectly connected through the virtual entity of joint formula, whereas the virtual entity is treated as a formula entity when the relationship is instantiated.

Other interentity relationships, interentity relationships between entity combinations, and relationships between entities and entity combinations in the combination of entities based on syndrome differentiation and treatment are instantiated according to different types of interentity relationships in the mode layer. For example, in the combination of syndrome differentiation and treatment, the relationship between entities in the combination of “therapeutic principle and the method of treatment” and “formula” entities is “use;” it has the same relationship with the same type of entities. The dose value of herbs is regarded as the attribute of “composition is,” “basic formula plus,” and “basic formula minus” in the relationship between herbs and formula or joint formula. There is no entity relationship connection between entity combinations of syndrome differentiation and treatment.

Other entities in the combination of non-10 main types of entities and syndrome differentiation and treatment entities are determined to have relations with some adjacent entities of specific types or with all entities in the whole combination of syndrome differentiation and treatment according to their relative positions with 10 main types of entities and corresponding combinations of syndrome differentiation and treatment. For example, time entities are generally related to their adjacent diseases, symptoms, etiology, and pathogenesis or used as the basis for addition and subtraction, whereas physicians and ancient Chinese medicine books entities are related to all entities in the adjacent combination of syndrome differentiation and treatment.

The third stage involves making full use of other data resources to supplement and improve the relationships and entities of those mentioned in the articles but with incomplete information. We obtained relevant information from the “Information Table of TCM Ancient Books” and the “Biographical Knowledge Base of Ancient Chinese Physicians” through the Internet. At the final stage of RE, we can supplement the alias, Dynasty, place of birth and death or activity of the doctors in the articles, year of completion, article ID, and other relationship information of the medical books. The synonym relation supplement mentioned in Fig. 1 refers to the standardization results to be reported subsequently.

### Standardization

Drawing on the “Clinic terminology of traditional Chinese medical diagnosis and treatment disease,” and dictionary of TCM syndrome and disease names, TCM dictionary, TCM textbooks, and online database, we built our own TCM synonyms list.

Special naming methods such as abbreviations, aliases, disorderly combinations of multiple elements, and adding modifiers to distinguish different states of the same thing often exist in ancient Chinese medicine books. If the extracted entities are not standardized, there will be many identical entities with different names and seemingly unrelated relationships in the constructed knowledge map, which is not conducive to the query and application of knowledge. Based on applying our TCM synonym list, we continued to use the manual + rule method for entity standardization. Examples are below.

#### Entities of Diseases and Symptoms

The synonym list was used for standardization, and then the entities that the synonym list could not cover were manually analyzed, and data and experts were consulted to determine their standard names. The manual standardization was conducted in our text annotation software.

#### Tongue Manifestation and Pulse Condition Entities

We standardized them by element decomposition and reorganization. For example, for a tongue-like entity, we first extracted the keywords according to the following six elements: tongue spirit, tongue color, form of the tongue, motility of the tongue, fur color, and texture of fur, and then reorganized the nouns in the above order. Finally, we added the modifiers containing the position and degree contained in the original entity before the reorganized nouns to obtain the standard name of the tongue-like entity.

#### Herbs Entities

The nature, taste, meridian entry, and efficacy of the same herbs with the same origin change after processing. Generally, two modifiers, “raw” and “cooked,” are used to distinguish whether the herbs have been processed. When modifiers are added, they represent two different entity types. However, entities in which we could not judge the type of herb from the abbreviation of TCM were truthfully entered. To illustrate, for “Shao(芍)” in the article, it is impossible to determine whether it is “paeoniae radix rubua” or “paeoniae radix alba.”

If all the entities extracted from the original text are replaced with standard words and set synonyms as the properties of standard words, the original expression of the entity is unobtainable through query. To retain the original meaning of ancient books and documents and avoid semantic loss as much as possible, we used the standard words and synonyms obtained from the above processing to update and expand the self-built synonym list and finally convert the relationship between the standard words and synonyms into triplet <synonyms, the standard word is, standard words > , which are stored in the knowledge map.

### Knowledge Graph Construction

The entities extracted from the ancient TCM books, and the entity relationships extracted through our three-stage RE method, as well as the supplementary ancient physician information, ancient TCM book information, and the updated synonym list after standardized processing, were processed into documents in acceptable format by the graph database software neo4j. Furthermore, they were imported into neo4j in batch for storage.

## Results

### Result Statistics

The results include the schema layer we built, the extracted entities and relationships, and the synonym list updated by the standardized operation ( Tables 1 2 3 4 ). Table 1 shows the number of relationships among the 10 main entity types and partner entity types defined in the pattern layer, including the total number of relationships when such entities are the subject and object of the relationship. After entity recognition and manual supplementary annotation, a total of 291,252 entities in 20 categories were extracted, whereas 48,036 entities were removed. Table 2 shows the entity extraction results of 10 main entity types. Table 3 represents a comparison of the RE results using our proposed method based entirely on the schema layer and shows the number of virtual entities of the joint formula and co-occurrence relationships. Table 4 shows the number of entity standard names in the updated thesaurus.

**Table 1:** Relationship quantity of the 10 main entity types and partner entity types

Entity type	Number of relationships	Number of relationship types
Disease	19	14
Symptom	23	14
Tongue manifestation	20	12
Pulse condition	20	12
Cause and mechanism of disease	34	15
Location of disease	15	8
Nature of disease	15	8
Therapeutic principle and method	32	10
Herb	30	10
Formula	31	11
Joint formula	40	7

**Table 2:** Entity extraction results of the 10 main entity types

Entity type	Number of extracted entities	Number of entities after weight removal
Disease	13,613	2,237
Symptom	32,935	10,134
Tongue manifestation	1,527	674
Pulse condition	5,687	2,527
Cause and mechanism of disease	47,038	13,934
Location of disease	14,025	618
Nature of disease	44,691	2,828
Therapeutic principle and method	19,231	5,670
Herb	68,430	3,830
Formula	10,965	2,060

**Table 3:** Results of the two relationship extraction methods

Including entity/relationship quantity	This research method	Full connection by mode layer (without joint formula)
Number of entities	81,955	71,914
Number of relationships	1,133,786	1,155,887
Number of joint formula virtual entities	10,041	–
Number of virtual entity relationships of a joint formula	152,845	–
Co-occurrence relationship quantity	102,936	–
Number of entities excluding the virtual entity of joint formula	71,914	–
Number of relationships excluding the virtual entity of the joint formula co-occurrence relationship	878,005	–

**Table 4:** Number of standard names in the thesaurus

Serial number	Entity type	Standard name quantity	Serial number	Entity type	Standard name quantity
1	Disease	1,640	7	Nature of disease	41
2	Symptom	768	8	Therapeutic principle and method	1,403
3	Tongue manifestation	38	9	Herb	1,678
4	Pulse condition	903	10	Formula	5,712
5	Cause and mechanism of disease	1,987	11	Title of TCM ancient books	1,176
6	Location of disease	52	12	Physician	538

### Evaluation

#### Entity Recognition Evaluation

We fine-tuned the pretrained models ERNIE/BERT, ERNIE/BERT + CRF, and ChatGLM3-b with the same annotation data and compared their performance in the average accuracy, average recall rate, and average F1 value of evaluation indicators. We further used the same test data to predict entity recognition in GPT3.5 of the open AI, ChatGLM3.0, and ChatGLM4.0, of Tsinghua University. The results are shown in Table 5 . Without combining with CRF, the testing effect of ERNIE is worse than BERT. But when combined with CRF, the testing effect of ERNIE is better than that of BERT. The entity recognition test results of the fine-tuned ChatGLM3-b, GPT3.5, ChatGLM3.0, and ChatGLM4.0 are also worse than those of the fine-tuned ERNIE and BERT. The reason for the poor accuracy may be that the annotation data of some entity types is sparse, and the word formation of some entity types is too complex and changeable, whereas the rules and features are too implicit, preventing the model from accurately identifying the boundaries of entities.

**Table 5:** Named entity recognition evaluation of the pretrained model

Model	Accuracy (average)	Recall rate (average)	F1 value (average)
ERNIE + CRF	0.74	0.80	0.77
ERNIE	0.36	0.51	0.42
BERT +CRF	0.72	0.77	0.74
BERT	0.71	0.76	0.73
ChatGLM3–6b	0.34	0.23	0.26
ChatGLM3.0	0.45	0.45	0.40
ChatGLM4.0	0.43	0.44	0.40
GPT3.5	0.35	0.50	0.38

Abbreviations: BERT, Bidirectional Encoder Representation from Transformers; CRF, Conditional Random Fields; ERNIE, Enhanced Representation through Knowledge Integration.

#### Relationship Extraction Evaluation

Table 3 shows the comparison between the extraction results of our method and the results of RE completely based on the pattern layer. The RE method we proposed extracted a total of 10,041 virtual entities from ancient books, 102,936 co-occurrence relationships, and 23,878 entities such as the ID of supplementary provisions and medical records. After removing the virtual entity relationships and co-occurrence relationships, the number of relationships was 277,882 less than the number of relationships extracted entirely based on the model layer. Hence, our method reduced the invalid entity relationships by approximately 24.04% compared with the RE based on the schema layer. Moreover, it had a significant inhibitory effect on the connection of invalid relationships. In addition, our method added 255,781 co-occurrence relationships and entity relationships of joint parties (an increase of 29.13%), which provides evidence for a more accurate and helpful understanding of knowledge.

**Fig. 2:**
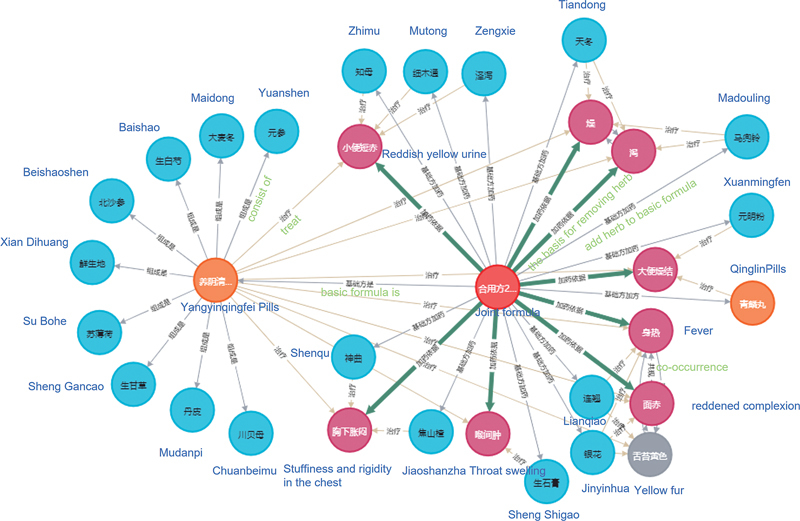
Test results of entity combination and their relationship instantiation based on a joint formula.

**Fig. 3:**
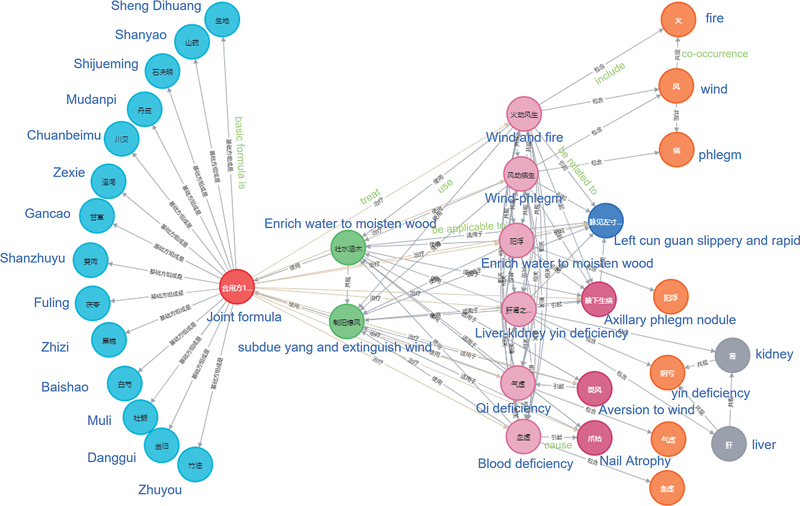
Test results of entity combination and relationship instantiation based on syndrome differentiation and treatment.

Figs. 2 and 3 show the test results of entity combination and relationship instantiation based on the proposed two frameworks. Fig. 2 is the result of entity combination and relationship instantiation of the partner, whereas Fig. 3 is the result of dialectical entity combination and relationship instantiation. It is demonstrated that our method can determine the relationship and structure between entities involved in the process of TCM dialectical treatment, which is beneficial for understanding and restoring the original meaning of the text description of ancient books from the knowledge map.

#### Knowledge Graph Evaluation

**Fig. 4:**
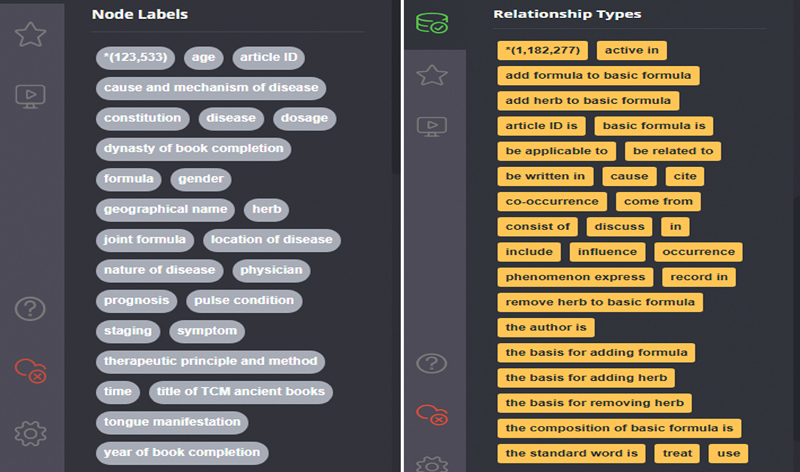
Neo4j graph database startup interface.

After importing the knowledge into the neo4j diagram database, we initiated the database, as shown in Fig. 4 . The knowledge map of ancient books with Yin deficiency syndrome contains 123,533 nodes and 1,182,277 relationships (including entities and relationships extracted from ancient books, ancient physicians and book information, and the synonym list).

**Fig. 5:**
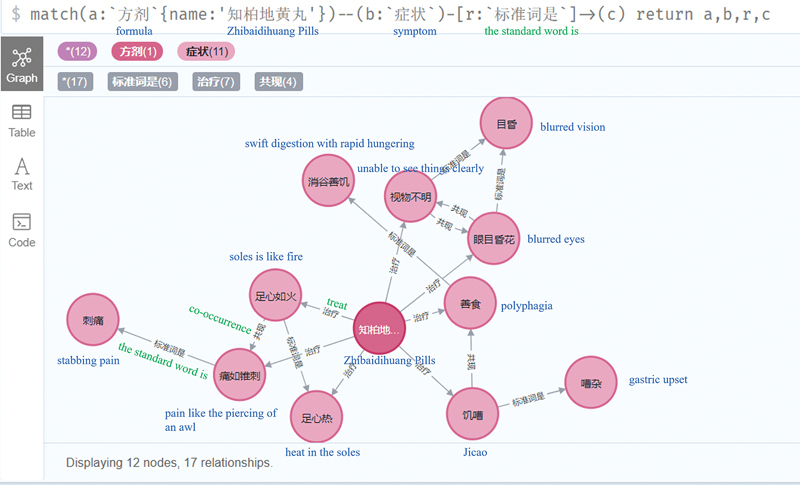
Relationship between Zhibai Dihuang wan and symptoms.

The nodes and relationships can be queried and operated in the database through the cipher statement, as shown in Fig. 5 . By querying the associated symptoms and symptom standard names of a prescription entity “Zhi bai di huang wan (知柏地黄丸),” we can obtain the specific symptoms that it can treat and their labeled names. In addition, we can find that some symptoms have the same standard names. Some symptoms appearing in the same combination of syndrome differentiation and treatment have a co-occurrence relationship. This is critical to the correct use of the KG relationship to assist diagnosis and treatment.

## Discussion

### Characteristics of the Knowledge Graph Model Layer in Ancient Books with Syndrome

The knowledge of ancient TCM books is complex and scattered, and the terminology is diverse. The correct schema layer design can structurally express and relate the highly complex diagnosis and treatment thinking and process contained in the knowledge of ancient Chinese medicine syndrome books, providing an interpretable reasoning path for building an intelligent system for differentiating syndromes. However, existing TCM KGs are mostly based on disease, whereas the syndrome KGs are limited and mostly constructed using modern literature as the data source. The literature review showed that syndrome is a concept determined only in modern times. There are few descriptions of “XX syndrome” in ancient TCM books related to Yin deficiency syndrome. However, the mechanism of Yin deficiency is exceptionally rich, whereas the mechanism of the disease is the essence of getting ill, and the syndrome is the external manifestation of the disease mechanism. Therefore, we set the entity type of the disease mechanism in the mode layer without the syndrome entity type. The discussion of the cause and mechanism of disease in TCM ancient books is variable, and it is difficult to identify and standardize them. Therefore, we selected the two entity types of nature of disease and location of disease to facilitate entity standardization. In addition, In clinical practice, formulas are often modified or combined based on basic prescriptions according to different situations. Therefore, we selected the virtual entity type of a joint formula in the model layer to emphasize the joint effect of the combination treatment regimen, which includes herb groups, joint use, and addition and subtraction of formula on other entities. The design of the joint formula reflects the importance of individualization in TCM diagnosis and treatment. It transforms the experience of changing TCM formulas for syndromes in ancient books into a computable and inferential logical chain, which can support dynamic formula adjustment in clinical decision-making.

### Recognition Method of Complex Structure Entity

Compared with the NER results of multiple pretrained models, although the knowledge coverage of large models is wider, there is still insufficient learning and understanding in the field of TCM ancient books. Moreover, LLMs with parameter compression are unable to add CRF output layers for the nontargeted training of entity recognition tasks. Even with fine-tuned of entity recognition tasks, the NER results are not as good as ERNIE + CRF and BERT + CRF. Therefore, we ultimately chose to use the fine-tuning model of ERNIE + CRF as the main model for entity recognition.

In addition, the F1 value of entity recognition of TCM, prescription, physician name, gender, tongue, pulse, and other types is higher, whereas the recognition effect of entity recognition of symptoms, etiology and pathogenesis or syndrome, disease, and treatment is poor. However, it is not only related to the sparsity of some types of entity data in the training data but also strongly related to the word formation complexity and length of entity text. For example, although the word length of the tongue and pulse entities is long, the word formation elements are relatively fixed, and the training effect is relatively good. However, the word length of the etiology and pathogenesis entities is not fixed, whereas the word formation elements are variable and contain a variety of modifiers. Consequently, it was the entity type with the worst recognition effect. However, the recognition effect can be significantly improved (F1 increased by more than 0.5) by decomposing it into a disease element and disease location element. Therefore, the definition of entity type is crucial for the effect of entity recognition. Entity types with complex word formation should be decomposed into entity types with fewer elements as much as possible.

### Constraint Relation Extraction Technology

Ancient TCM books not only describe the language concisely but also according to the process of syndrome differentiation and treatment, the main types of entities in the ancient books' fixed-relationship-type restrictions, the scope of the relationship, the order of occurrence, and even the same two types of entities. The relationship in different scenarios is also different. This work adds entity constraint relationships, which can avoid a large number of connections that do not conform to the semantics of the original text, while maintaining the author's original intention, providing more accurate knowledge for TCM clinical decision-making systems. In addition, to retain more potential information from the original text and help professionals in the field better understand the original idea of the author, we added the virtual entity type of a “joint formula.” These characteristics and designs limit the possibility of using deep learning in our research or reduce the application effect of deep learning. However, too many interference factors in the text will also reduce the accuracy of the rule-based method. In future research, we will combine the two to design a more efficient, accurate, and consistent RE method.

## Conclusion

We combined the characteristics of ancient TCM books and modern literature and summarized the work of scholars in the field of TCM knowledge mapping and natural language processing, analyzing the available methods and technologies and their advantages and disadvantages.

Using modern information means to store TCM knowledge in the form of a KG is more conducive to the dissemination, preservation, and innovative development of TCM. The KG can help professionals in the field better understand the knowledge of ancient books and learn and master TCM theory and practice. Therefore, we successfully constructed a KG of ancient TCM books on Yin deficiency syndrome using TCMSF and improved the accuracy of the knowledge and relationship expression.

Our future research will study knowledge extraction technology using ancient TCM books, improve extraction efficiency and accuracy, and apply these technologies to the knowledge management of more TCM subfields.

## References

[1] Guo M Y Zhou L Sun Y The application of “domain ontology seven step method” in the construction of traditional Chinese medicine syndrome differentiation and reasoning knowledge base world science and technology Modern Trad Chin Med 2019 12 2646 2651

[2] Zhou H Peng F L Wei C F [Research and practice on the construction of a knowledge graph for diagnosis and differentiation of traditional Chinese medicine] Journal of Medical Informatics 2020 12 41 44

[3] Zhang M Z Yang Z G Liu C Fang L Traditional Chinese medicine knowledge service based on semi-supervised BERT-BiLSTM-CRF Model2020 International Conference on Service Science (ICSS), Xining, China.2020 64 69

[4] Sun Y Zhao Z Wang Z Leveraging a joint learning model to extract mixture symptom mentions from traditional Chinese medicine clinical notes BioMed Res Int 2022 2022 2.146236E6 10.1155/2022/2146236 PMC8923793 35299894

[5] Chen K Wang W Cai J TRBNER: named entity recognition of TCM medical records based on multi-feature fusion IET Conf Proc 2025 2024 21 174 181

[6] Jiang M Sanger T Liu X Combining contextualized embeddings and prior knowledge for clinical named entity recognition: evaluation study JMIR Med Inform 2019 7 04 e14850 31719024 10.2196/14850 PMC6913757

[7] Bao Z S Song B Y Zhang W B [Named entity recognition in traditional Chinese medicine books combining semi-supervised learning and rule-based approach.] J Chinese Information Processing 2022 6 90 100

[8] Song Z Xu W Liu Z Chen L Su H A BERT-based named entity recognition method of warm disease in traditional Chinese medicineIn: 2023 IEEE 18th Conference on Industrial Electronics and Applications (ICIEA) Ningbo, China: IEEE;2023 1226 1231

[9] Zhang W Wu Z Song G Huo Q Wang B [named entity recognition of traditional Chinese medicine classics based on SiKuBERT and multivariate data embedding] J South China Univ Technol Nat Sci Ed 2024 52 06 128 137

[10] Tuo M M Yang W Z Review of entity relation extraction J Intell Fuzzy Syst 2023 44 05 7391 7405

[11] Wang X T Miao F Liu H X Zhang G T Jin L B Joint extraction of entities and relations from ancient Chinese medical literatureInternational Conference on Culture-Oriented Science & Technology (ICCST).2021 369 372

[12] Jiang S Li Z Zhao H Ding W Entity-relation extraction as full shallow semantic dependency parsing IEEE/ACM Trans Audio Speech Lang Process 2024 32 1088 1099

[13] Zhang T Huang Z Wang Y Wen C Peng Y Ye Y Information extraction from the text data on Traditional Chinese Medicine: a review on tasks, challenges, and methods from 2010 to 2021 Evid Based Complement Alternat Med 2022 2022 1.679589E6 10.1155/2022/1679589 PMC9122692 35600940

[14] Liu W Yin M Zhang J Cui L A joint entity relation extraction model based on relation semantic template automatlyical constructed Comput Mater Continua 2024 78 01 975 997

[15] Hu J Tang B Lyu N He Y Xiong Y CMBEE: a constraint-based multi-task learning framework for biomedical event extraction J Biomed Inform 2024 150 104599 38272433 10.1016/j.jbi.2024.104599

